# *Notes from the Field*: Increase in Meningococcal Disease Among Persons with HIV — United States, 2022

**DOI:** 10.15585/mmwr.mm7224a4

**Published:** 2023-06-16

**Authors:** Amy B. Rubis, Rebecca L. Howie, Daya Marasini, Shalabh Sharma, Henju Marjuki, Lucy A. McNamara

**Affiliations:** 1 Division of Bacterial Diseases, National Center for Immunization and Respiratory Diseases, CDC.

Meningococcal disease, caused by the bacterium *Neisseria meningitidis*, is a sudden-onset, life-threatening illness that typically occurs as meningitis or meningococcemia. The most common signs and symptoms of meningitis include fever, headache, and stiff neck; the most common signs and symptoms of meningococcemia are fever, chills, fatigue, vomiting, diarrhea, cold hands and feet, and severe aches or pain.* Quadrivalent meningococcal conjugate vaccination (MenACWY) is routinely recommended for adolescents and persons at increased risk for meningococcal disease (*1*), including those with HIV. In 2016, a 2-dose series of MenACWY was recommended by the Advisory Committee on Immunization Practices (ACIP) for persons with HIV and incorporated into the U.S. immunization schedule. Coverage among persons with HIV, however, remains low: in a study of administrative claims data during January 2016–March 2018, only 16.3% of persons with HIV received ≥1 doses of MenACWY vaccine within 2 years after their diagnosis (*2*). This report describes an increase in meningococcal disease among persons with HIV in the United States in 2022. Data are typically finalized in the fall of the next year; therefore, this report is based on preliminary data for 2022.

Meningococcal disease cases are reported through the National Notifiable Diseases Surveillance System, with additional epidemiologic information and isolates obtained through Enhanced Meningococcal Disease Surveillance. Isolates are characterized using whole genome sequencing to determine serogroup and molecular typing information. This activity was reviewed by CDC and was conducted consistent with applicable federal law and CDC policy.^†^

During 2017–2021, five to 15 meningococcal disease cases were reported each year among persons with HIV, representing 1.5%–4.3% of all meningococcal disease cases annually (Figure). Based on preliminary data, 29 meningococcal disease cases have been reported among persons with HIV in 2022, accounting for 9.8% of all cases. This case count might increase when reporting is complete.

**FIGURE:**
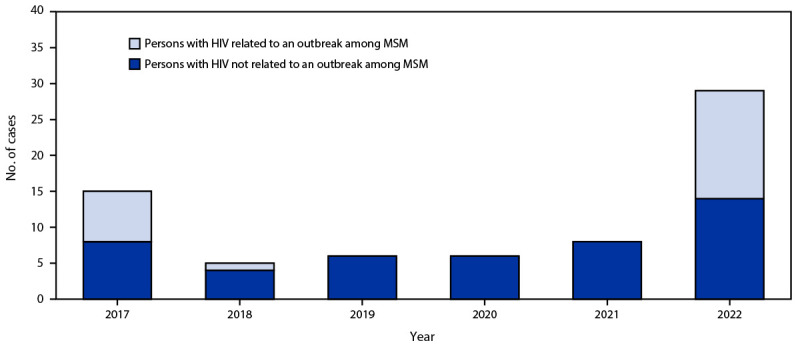
Meningococcal disease cases among persons with HIV, by year — United States, 2017–2022* **Abbreviation:** MSM = men who have sex with men. * Data for 2022 are not yet final, and these numbers might increase when reporting is complete for the year.

Among the 29 meningococcal disease cases among persons with HIV in 2022, 22 had not received MenACWY vaccine, six had unknown MenACWY vaccination history, and one had received MenACWY vaccine, but the number of doses received was unknown. Fifteen of the 29 cases were part of a large serogroup C outbreak that occurred primarily among men who have sex with men (MSM); however, after excluding MSM outbreak-associated cases for all years, a substantial increase in meningococcal disease cases among persons with HIV in 2022 remained (i.e., 14 cases compared with four to eight cases per year during 2017–2021) (Figure). Of the 14 cases among persons with HIV in 2022 that were not related to the outbreak primarily among MSM, nine were caused by a single strain of *N. meningitidis* serogroup Y clonal complex CC174 sequence type ST-1466. Eight of these nine cases occurred in Black or African American persons, and seven occurred among MSM. The nine cases caused by a single strain were reported from three states with no identified connections among cases. The remaining five cases were not clustered geographically and had no identified epidemiologic connections.

MenACWY vaccine coverage among persons with HIV is low; given the recent increase in meningococcal disease cases in this population, health care providers should ensure that all persons with HIV are up to date with MenACWY vaccination per ACIP recommendations, as well as other vaccines recommended for this population. Health care providers should also maintain a high index of suspicion for meningococcal disease among persons with HIV who have symptoms of meningococcal disease. CDC recommends that all persons be screened for HIV at least once in their lifetime (*3*). Providers should ensure that patients with meningococcal disease and unknown HIV status are screened for HIV.

## Footnotes

*
https://www.cdc.gov/meningococcal/about/symptoms.html

† 45 C.F.R. part 46.102(l)(2), 21 C.F.R. part 56; 42 U.S.C. Sect. 241(d); 5 U.S.C. Sect. 552a; 44 U.S.C. Sect. 3501 et seq.
